# Comparison Between Blood-Brain Barrier Water Exchange Rate and Permeability to Gadolinium-Based Contrast Agent in an Elderly Cohort

**DOI:** 10.3389/fnins.2020.571480

**Published:** 2020-11-30

**Authors:** Xingfeng Shao, Kay Jann, Samantha J. Ma, Lirong Yan, Axel Montagne, John M. Ringman, Berislav V. Zlokovic, Danny J. J. Wang

**Affiliations:** ^1^Laboratory of FMRI Technology (LOFT), USC Mark & Mary Stevens Neuroimaging and Informatics Institute, Keck School of Medicine, University of Southern California, Los Angeles, CA, United States; ^2^Department of Neurology, Keck School of Medicine, University of Southern California, Los Angeles, CA, United States; ^3^Zilkha Neurogenetic Institute and Department of Physiology and Neuroscience, Keck School of Medicine, University of Southern California, Los Angeles, CA, United States

**Keywords:** blood-brain barrier (BBB), water exchange rate, permeability, arterial spin labeling (ASL), white matter hyperintensity (WMH), cerebral small vessel disease (CSVD)

## Abstract

**Background:** Dynamic contrast-enhanced (DCE) MRI using intravenous injection of gadolinium-based contrast agents (GBCAs) is commonly used for imaging blood-brain barrier (BBB) permeability. Water is an alternative endogenous tracer with limited exchange rate across the BBB. A direct comparison between BBB water exchange rate and BBB permeability to GBCA is missing. The purpose of this study was to directly compare BBB permeability to GBCA (Ktrans and k_Gad_ = Ktrans/Vp) and water exchange rate (kw) in a cohort of elderly subjects at risk of cerebral small vessel disease (cSVD).

**Methods:** Ktrans/k_Gad_ and kw were measured by DCE-MRI and diffusion prepared pseudo-continuous arterial spin labeling (DP-pCASL), respectively, at 3 Tesla in 16 elderly subjects (3 male, age = 67.9 ± 3.0 yrs) at risk of cSVD. The test-retest reproducibility of kw measurements was evaluated with repeated scans ~6 weeks apart. Mixed effects linear regression was performed in the whole brain, gray matter (GM), white matter (WM), and 6 subcortical brain regions to investigate associations between Ktrans/k_Gad_ and test-retest kw. In addition, kw and Ktrans/k_Gad_ were compared in normal appearing white matter (NAWM), white matter hyperintensity (WMH) lesions and penumbra.

**Results:** Significant correlation was found between kw and Ktrans only in WM (β = 6.7 × 10^4^, *P* = 0.036), caudate (β = 8.6 × 10^4^, *P* = 0.029), and middle cerebral artery (MCA) perforator territory (β = 6.9 × 10^4^, *P* = 0.009), but not in the whole brain, GM or rest 5 brain regions. Significant correlation was found between kw and k_Gad_ in MCA perforator territory (β = 1.5 × 10^3^, *P* = 0.049), medial-temporal lobe (β = 3.5 × 10^3^, *P* = 0.032), and hippocampus (β = 3.4 × 10^3^, *P* = 0.038), but not in the rest brain regions. Good reproducibility of kw measurements (ICC=0.75) was achieved. Ktrans was significantly lower inside WMH than WMH penumbra (16.2%, *P* = 0.026), and k_Gad_ was significantly lower in NAWM than in the WMH penumbra (20.8%, *P* < 0.001).

**Conclusion:** kw provides a measure of water exchange rate across the BBB with good test-retest reproducibility. The BBB mechanism underlying kw and Ktrans/k_Gad_ is likely to be different, as manifested by correlations in only three brain regions for each pair of comparison between kw and Ktrans or k_Gad_.

## Introduction

The blood-brain barrier (BBB) plays important roles in regulating the exchange of substances between blood and brain parenchyma, and protecting the central nervous system (CNS) from neurotoxic substances circulating in the blood (Sweeney et al., 2019). Trans-endothelial permeability to large plasma proteins and inorganic solutes is low in healthy brain tissue because of endothelial cell membrane and tight junctions (Nitta et al., 2003; Wardlaw et al., 2003). The BBB becomes increasingly permeable to large molecules with advancing age, particularly in patients with vascular dementia and cerebral small vessel disease (cSVD) (Farrall and Wardlaw, 2009; Montagne et al., 2015), while water exchange rate across the BBB shows a reverse trend and declines with aging (Li et al., 2005; Anderson et al., 2020).

Dynamic contrast-enhanced MRI (DCE-MRI), which uses gadolinium-based contrast agents (GBCAs), has been widely used to study a broad spectrum of CNS disorders associated with BBB disruption, including multiple sclerosis (MS), ischemic stroke, and brain tumor (Heye et al., 2014; Montagne et al., 2016). Recently increasing DCE-MRI studies are being conducted to evaluate the subtle change of BBB leakage rate to GBCAs (or Ktrans) in pathologies such as cSVD, diabetes, and Alzheimer's Disease (AD) (Starr et al., 2003; Montagne et al., 2016, 2017; Zhang et al., 2017; Nation et al., 2019). There is potential Gd deposition in the brain of persons undergoing repeated contrast-enhanced MRIs at 3–4 month intervals such as MS patients, but not in older individuals undergoing DCE MRI on a yearly basis (Gulani et al., 2017; Montagne et al., 2019). Since contrast agents have relatively large molecular weights (Gd-DTPA 550 Da), BBB permeability has to reach a critical level before extravasation occurs (Nitta et al., 2003).

Alternative to contrast agents, water is an endogenous tracer, and has much smaller molecular weight (~18 Da). A recent review comprehensively summarized mechanisms of water exchange across the BBB, up-to-date acquisition methods and mathematical models (Dickie et al., 2020). Trans-endothelium water exchange is through both passive (diffusion) and active (i.e., cotransport by ion pumps, specialized carrier-medicated proteins, and transcytosis) pathways. Specifically, water exchange is facilitated by dedicated water channel, aquaporin-4 (AQP4), at the astrocyte-endfoot between perivascular and interstitial space (Ibata et al., 2011; Papadopoulos and Verkman, 2013; Ohene et al., 2019; Dickie et al., 2020). Assessing BBB water exchange could provide a more sensitive assessment of BBB dysfunction at the early stage of disease progression. Arterial spin labeling (ASL) is a non-invasive technique to measure cerebral blood flow (CBF), and kinetic models have been proposed to map the transvascular water exchange rate based on the T2 (Ohene et al., 2019) or diffusion coefficient (Shao et al., 2019) differences between the intra- and extravascular compartments. Regional water exchange across BBB can be quantified based on the kinetic modeling of ASL signals in the two compartments. Clinical studies have shown that altered BBB water exchange is associated with aging (Li et al., 2005; Anderson et al., 2020), multiple sclerosis (Rooney et al., 2015), and obstructive sleep apnea (Palomares et al., 2015). In addition, a recent preclinical study reported that trans-BBB water exchange is increased in AD rats, while GBCA permeability did not differ between AD and wild type rats (Dickie et al., 2019), in contrast to several other studies in rodent models of AD (Montagne et al., 2017). Although a number of studies on BBB water exchange have been conducted, a direct comparison between BBB water exchange rate and BBB permeability to GBCA in humans is missing.

The purpose of this study was to directly compare BBB permeability to GBCA (volume transfer constant or leakage rate: Ktrans and exchange rate of GBCA: k_Gad_) measured by DCE-MRI and water exchange rate (kw) across the BBB measured by diffusion-prepared pseudo-continuous arterial spin labeling (DP-pCASL) (St Lawrence et al., 2012; Shao et al., 2019) in a cohort of elderly subjects at risk of cSVD. Correlation analysis was performed in the whole brain, gray and white matter, and 6 subcortical brain regions to investigate associations between the two measurements of kw and Ktrans. The test-retest reproducibility of this DP-pCASL sequence was evaluated with repeated scans ~6 weeks apart. In addition, the two BBB measurements were compared in normal appearing white matter (NAWM), lesion and penumbra of white matter hyperintensity (WMH), which is generally considered as a surrogate imaging marker of cSVD.

## Materials and Methods

### Human Subjects

Sixteen aged subjects (3 male, age = 67.9 ± 3.0 yrs, all Latinx) enrolled in the MarkVCID study (www.markvcid.org) underwent both DCE-MRI and DP-pCASL scans were included and analyzed in the current study. Clinical evaluation of the participants was performed by a board-certified neurologist (JMR) using the Clinical Dementia Rating (CDR) scores. The CDR is a structured interview of the subject and informant based on which subjects were rated as: 0 (asymptomatic), 0.5 (equivocal impairment), 1 (mild), 2 (moderate), or 3 (severe dementia). In this study, all subjects were rated as CDR 0 or 0.5. The subject inclusion criteria were: (1) Fluency in Spanish and/or English; (2) Age > 60 yrs; (3) Have capacity for and sign consents indicating so, or give assent and have an appropriate surrogate (as determined by California law) to sign consent; (4) For demented subjects, have an appropriate informant who is also willing and able to accompany the subject. Non-demented subjects must also have an informant willing to participate by phone; (5) Previous diagnosis of diabetes mellitus, hypertension, and/or hypercholesterolemia (high-risk group); (6) Healthy subjects without previous diagnosis of diabetes mellitus, hypertension, or hypercholesterolemia (low risk group). The exclusion criteria included history of prior clinical stroke, head trauma, contraindications to MRI, abnormal renal function, other concurrent neurologic or psychiatric illnesses or abnormal structural MRI (e.g., mass lesions, cystic infarction, etc.). All subjects were required to refrain from caffeine intake and nicotine use 3 h prior to and during the study visit. The demographic characteristics of the recruited subjects are exhibited in Table 1. Based on the inclusion criteria, 2 of the 16 enrolled subjects belonged to the low risk group while the rest 13 belonged to the high-risk group (clinical record was not available in one subject).

**Table 1:** Demographic characteristics of 16 subjects recruited in this study.

		**Count**	**Column *N*%**
Sex	Male	3	18.8%
	Female	13	81.3%
Age range (years)	62–86		
Vascular risk factor	Diabetes	7 (of 15)	46.7%
	Hypertension	8 (of 15)	53.3%
	Hypercholesterolemia	12 (of 15)	80.0%
Global CDR scale	0 (Normal)	12	75.0%
	0.5 (Very mild dementia)	4	25.0%
Fazekas scale	≤1 (Mild WMH)	4	25.0%
	2 (Moderate WMH)	9	56.3%
	4 (Severe WMH)	3	18.8%

*Vascular risk factors (presence of diabetes, hypertension or hypercholesterolemia) was defined by a past diagnosis and/or current treatment for these conditions from 15 subjects (clinical record of one subject was missing). Neurocognitive performance was evaluated by global Clinical Dementia Rating (CDR) scale. Severity of white matter lesions were quantified by the total Fazekas scale, which is the sum of 2 scales rated from 0 (absent) to 3 (large confluent areas) in periventricular white matter and deep white matter*.

### MRI Experiments

MRI scans were performed on a Siemens 3T Prisma system (Erlangen, Germany) using a 20-channel head coil after subjects provided written informed consent according to a protocol approved by the Institutional Review Board (IRB) of the University of Southern California.

Water exchange across the BBB was measured by the DP-pCASL sequence with background suppressed 3D gradient- and spin-echo (GRASE) readout (Shao et al., 2019). Imaging parameters were: Field-of-view (FOV) = 224 mm, matrix size = 64 × 64, 12 axial slices (10% oversampling), resolution = 3.5 × 3.5 × 8 mm^3^, turbo factor = 14, EPI factor = 64, bandwidth = 3,125 Hz/pixel, TE = 36.5 ms, TR = 4,000 ms, label/control duration = 1,500 ms, centric ordering, timing of background suppression was optimized according to Shao et al. (2018). A two-stage approach was utilized to measure arterial transit time (ATT) and water exchange rate (kw) (St Lawrence et al., 2012): Fifteen repetitions were acquired during the flow encoding arterial spin tagging (FEAST) scan at post-labeling delay (PLD) = 900 ms with a total acquisition time of 4 min for estimating ATT (Wang et al., 2003). kw was calculated from scans acquired at PLD = 1,800 ms, when the labeled blood reaches the microvascular compartment, with *b* = 0 and 50 s/mm^2^, respectively. Twenty repetitions were acquired for each *b*-value of the kw scan, and the total acquisition time was 6 min. CBF was quantified from perfusion signals acquired at PLD = 1,800 ms without diffusion preparation. Test and retest DP-pCASL scans were collected about 6 weeks apart to evaluate the reproducibility of the kw measurement.

BBB permeability to GBCA was measured by DCE-MRI, which consisted of a pre-contrast T1-mapping protocol and a dynamic T1-weighted gradient-echo acquisition. T1 map was estimated from 3D fast low angle shot (FLASH) images with 5 different flip angles: 2^0^, 5^0^, 10^0^, 12^0^, and 15^0^. Imaging parameters for T1 mapping were: FOV = 175 × 175 mm^2^, 14 oblique coronal slices through the hippocampus and basal ganglia as well as carotid arteries for individual arterial input function (AIF), resolution = 1.1 × 1.1 × 5 mm^3^, TE = 2.18 ms, TR = 5.14 ms. Imaging parameters for dynamic T1w acquisition were: FOV = 175 × 175 mm^2^, 14 slices, resolution = 1.1 × 1.1 × 5 mm^3^, TE = 3 ms, TR = 8 ms, temporal resolution = 15 s, 64 frames were acquired with a total acquisition time of 16 min. Contrast agent (Dotarem, Gadoterate meglumine, 0.5 mmol/mL) was injected after 30 s of image acquisition with an average injection rate of 3 mL/s. Dose of contrast agent was 0.2 mL/kg or 0.1 mmol/kg body weight. Each subject only underwent one DCE-MRI scan.

Slice orientation was different between DCE-MRI and DP-pCASL scans. The labeling plane was programmed to be parallel to the DP-pCASL imaging slab and axial orientation will maximize the labeling efficiency (perpendicular to feeding carotid arteries). DCE-MRI was acquired in coronal direction to minimize the inflow effects of arterial input function. When the body coil was used to excite a slab in the coronal orientation, the excitation extends in the superior-inferior direction down through the neck into the chest, which ensures that the carotids all the way down to the aortic arch were exposed to the RF pulses. Since both DCE-MRI and DP-pCASL were acquired with 3D readout, loss of information due to normalization should be negligible. T1-weighted MP-RAGE images (resolution = 1 × 1 × 1 mm^3^, sagittal acquisition, inversion time/TE/TR = 900/2.98/2300 ms) were acquired for co-registration and normalization of kw and Ktrans maps. T2-weighted fluid-attenuated inversion recovery (FLAIR) images (resolution = 1 × 1 × 1 mm^3^, inversion time/TE/TR = 1800/388/5000 ms) images were acquired for WMH segmentation.

### Data Analysis

**Figure 1:**
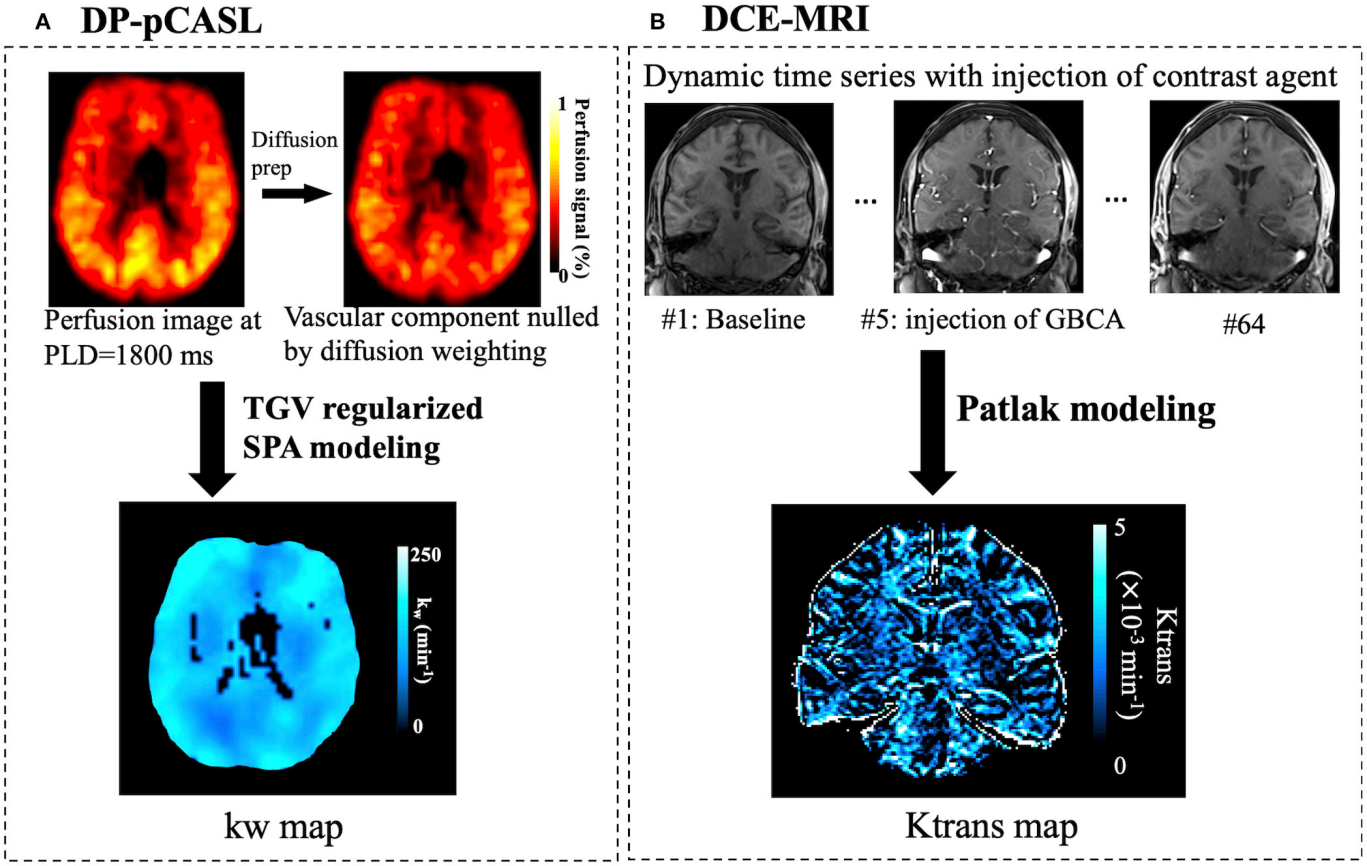
Processing pipelines for kw **(A)** and Ktrans **(B)** quantification. The first row in **(A)** shows perfusion images with and without diffusion preparation. The scale bar indicates the percentage perfusion signal relative to M0 signal. The vascular component of the ASL signal was nulled by a small diffusion weighting. The kw map was calculated by a total-generalized-variation (TGV) regularized single-pass-approximation (SPA) model using the tissue (or capillary) fraction of the ASL signal at the PLD of 1,800 ms as one of the inputs. Ktrans maps were fitted from dynamic DCE-MRI times series using the Patlak model.

DCE-MRI dynamic series and DP-pCASL control/label images were corrected for rigid head motion off-line using SPM12 (Well-come Trust Centre for Neuroimaging, UCL). Temporal fluctuations in the DP-pCASL image series owing to residual motion and physiological noise were minimized using principle component analysis (Shao et al., 2017). Figure 1 shows the processing pipeline for kw and Ktrans quantification. For DP-pCASL, the tissue (extra-vascular) and capillary (intra-vascular) compartments of the ASL signal were separated by a small diffusion gradient of 50 s/mm^2^. The differentiation between the two compartment is reliable given the large diffusion coefficient difference (~100-fold) between the intra- and extra-vascular compartments (Shao et al., 2019). kw map was calculated by a total-generalized-variation (TGV) (Spann et al., 2020) regularized single-pass-approximation (SPA) model (St Lawrence et al., 2012) using the tissue (or capillary) fraction of the ASL signal at the PLD of 1,800 ms, incorporating ATT, T1 of arterial blood [1.66 s (Lu et al., 2004)] and brain tissue as inputs for the algorithm (Shao et al., 2019). Voxel-wise tissue T1 map was fitted from background suppressed control images acquired at 2 PLDs (Shao and Wang, 2017). Ktrans and fractional plasma volume (Vp) maps were fitted by Patlak model (Heye et al., 2016) after noise filtering (voxel-wise, time domain moving average of 3 tissue concentration data points) using ROCKETSHIP software (Barnes et al., 2015; Montagne et al., 2015; Nation et al., 2019). Patlak model ignores the back flux from the extravascular into vascular compartment and is a suitable approach for measuring low-level BBB permeability (Barnes et al., 2016; Heye et al., 2016). AIF was determined from the carotid artery of each individual.

Volume transfer constant or leakage rate, Ktrans, is a composite parameter of CBF and permeability-surface product (PS). Under limited permeability condition (CBF >> PS) (Cuenod and Balvay, 2013), Ktrans approximately equals to PS. For a suitable comparison with water exchange rate kw, exchange rate of GBCA (k_Gad_) was computed as the ratio between Ktrans (PS) and fractional plasma volume Vp:

(1) $$\begin{array}{l} {\text{k}}_{\text{Gad}}=\frac{\text{Ktrans}}{\text{Vp}} \end{array}$$

**Figure 2:**
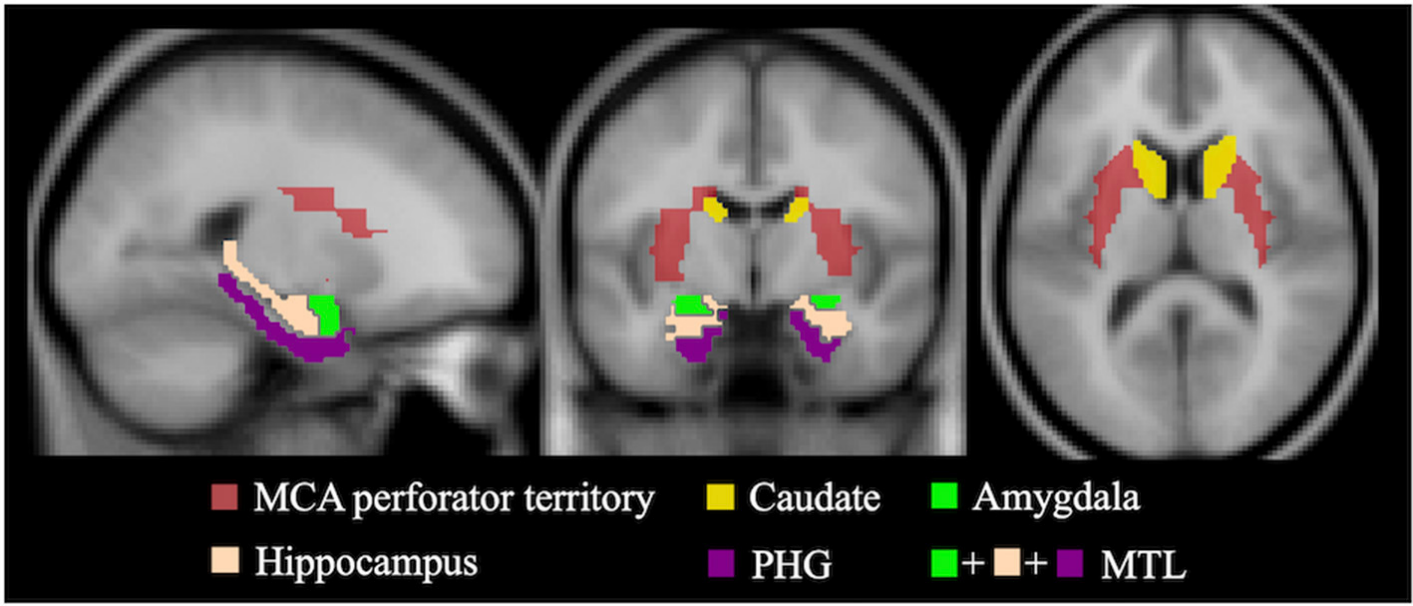
Template masks for regional Ktrans and kw measurement. ROIs of Hippocampus, PHG and amygdala were combined to form MTL. White matter and gray matter masks were not shown for better visualization of smaller ROIs. MCA perf, middle cerebral artery perforator territory; MTL, medial-temporal lobe; PHG, parahippocampal gyrus.

Ktrans, k_Gad_ and kw maps, together with aligned structural FLASH and GRASE images, were co-registered to T1-weighted MP-RAGE volumes, and then normalized into the canonical MNI space for regional analysis. Correlations between Ktrans/k_Gad_ and kw were studied in gray matter (GM), white matter (WM) and six subcortical regions of interests (ROIs): perforator territory of middle cerebral artery (MCA perf) (Wang et al., 2012), caudate, medial-temporal lobe (MTL) and subregions, including hippocampus, parahippocampal gyrus (PHG) and amygdala based on the AAL template (Tzourio-Mazoyer et al., 2002), as illustrated in Figure 2.

**Figure 3:**
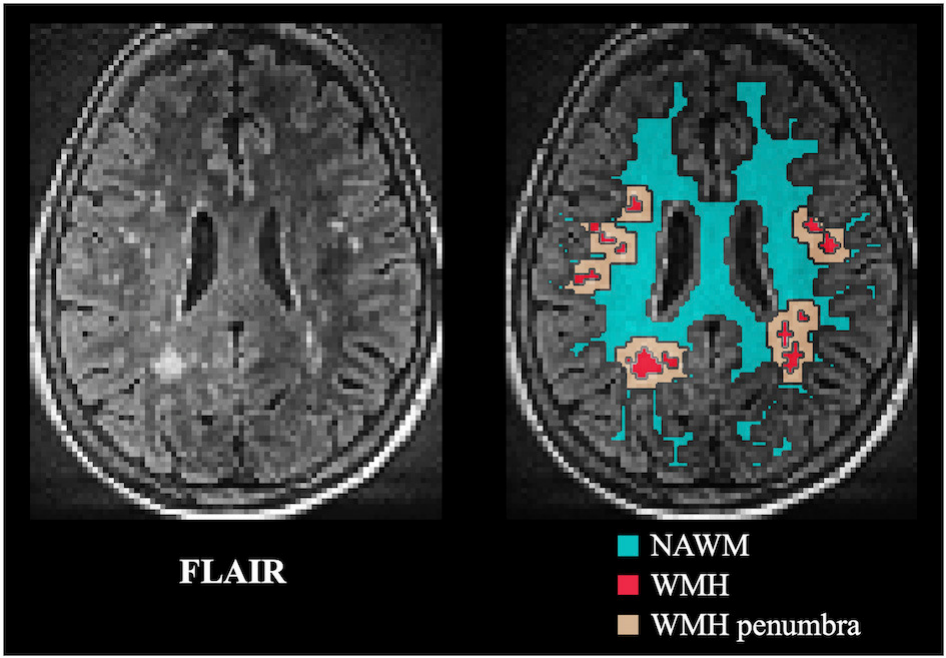
FLAIR image and overlaid template masks for NAWM, WMH and WMH penumbra (4-mm width). NAWM, normal appearing white matter; WMH, white matter hyperintensity.

Ktrans, k_Gad_ and 2-visit averaged kw, ATT and CBF were compared in regions of NAWM, WMH and WMH penumbra, respectively. WMH was manually segmented by two clinical fellows from FLAIR images (resolution = 1 × 1 × 1 mm^3^, inversion time/TE/TR = 1800/388/5000 ms) using ITK-SNAP (www.itksnap.org) (Yushkevich et al., 2006). WMH penumbra was defined as the border surrounding WMH with 4-mm width according to previous reports (Huisa et al., 2015). To improve the accuracy, WM mask was segmented from MP-RAGE and eroded (2 mm) to avoid potential partial volume effect from GM or cerebrospinal fluid (CSF). WMH and surrounding penumbra regions were excluded from WM mask to form NAWM mask, as illustrated in Figure 3. Four subjects with WMH volumes smaller than 1 cm^3^ were excluded for analysis.

The test-retest reproducibility of kw and CBF were quantified by intra-class correlation coefficient (ICC). Bland-Altman plots were generated to display the spread of data and to evaluate the agreement between test and retest kw measurements. Correlations between kw from both test and retest scans and Ktrans were evaluated using mixed effects linear regression model implemented in STATA 13.1 (College Station, Texas), incorporating time (test/retest) as the random variable. Pair-wise *t*-test was performed to evaluate the test/retest averaged kw, ATT, CBF and Ktrans/k_Gad_ in GM, WM, six subcortical regions and three WMH related regions (NAWM, WMH and WMH penumbra). Correlations with *P* < 0.05 were considered as significant (2-sided).

## Results

### Correlations Between Water Exchange Rate (kw), Exchange Rate of Contrast Agent (k_Gad_) and Ktrans

Average Ktrans, k_Gad_, Vp, and test-retest averaged kw, CBF, and ATT values were summarized in Table 2. Whole brain average Ktrans was 6.6 × 10^−4^ min^−1^, which falls into the range of normal appearing tissue and indicates the lack of severe BBB disruption (Heye et al., 2014, 2016). The highest BBB leakage to GBCA was found in the hippocampus (7.6 × 10^−4^ min^−1^), which was significantly higher than the whole-brain average Ktrans (*P* = 0.0002). Ktrans in other brain regions including GM (6.8 × 10^−4^ min^−1^, *P* = 0.003), WM (6.1 × 10^−4^ min^−1^, *P* = 0.003), and caudate (5.7 × 10^−4^ min^−1^, *P* = 0.001) were also significantly different from the whole-brain average (6.6 × 10^−4^ min^−1^), as indicated by † in Table 2. Whole brain average Vp was 3.5%, and Vp in GM (4.2%) was significantly higher than Vp in WM (1.9%) (*P* < 0.001). As a result, k_Gad_ in WM was 106% higher than GM (*P* < 0.0001). Ktrans in other brain regions including amygdala (1.2 × 10^−2^ min^−1^, *P* < 0.0001), hippocampus (1.1 × 10^−2^ min^−1^, *P* < 0.0001), PHG (1.7 × 10^−2^ min^−1^, *P* = 0.0027), MTL (1.3 × 10^−2^ min^−1^, *P* < 0.0001), MCAperf (1.5 × 10^−2^ min^−1^, *P* = 0.007), GM (1.6 × 10^−2^ min^−1^, *P* < 0.0001), and WM (3.3 × 10^−2^ min^−1^, *P* < 0.0001) were also significantly different from the whole-brain average (1.9 × 10^−2^ min^−1^), as indicated by † in Table 2.

**Table 2:** Summary of Ktrans, k_Gad_, test-retest averaged kw values, and regression coefficients of mixed-effect linear regressions between Ktrans/k_Gad_ and test-retest kw in the whole brain, gray matter, white matter, and six subcortical brain regions.

	**kw (min^**−1**^)**	**BBB permeability to contrast agent**				**Vp (%)**	**CBF (ml/100g/min)**	**ATT (ms)**
		**Ktrans (×10^**−4**^ min^**−1**^)**	**β: kw **~** Ktrans (*P*-value)**	**k_**Gad**_ (×10^**−2**^ min^**−1**^)**	**β: kw **~** k_**Gad**_ (*P*-value)**			
Whole brain	122.3 ± 16.5	6.6 ± 0.7	6.5 × 10^4^ (0.19)	1.9 ± 0.3	0.9 × 10^3^ (0.42)	3.5 ± 0.6	43.9 ± 5.1	1188.5 ± 113.1
Gray matter	122.6 ± 15.6	6.8 ± 0.7^†^	2.4 × 10^4^ (0.65)	1.6 ± 0.2^†^	0.8 × 10^3^ (0.54)	4.2 ± 0.7	44.8 ± 5.2	1183.0 ± 115.1
White matter	121.9 ± 17.2	6.1 ± 1.1^†^	6.7 × 10^4^ (0.036)*	3.3 ± 0.7^†^	0.1 × 10^3^ (0.81)	1.9 ± 0.5	37.2 ± 4.4	1230.7 ± 106.6
MCA perf	126.2 ± 21.5	6.3 ± 1.6	6.9 × 10^4^ (0.009)*	2.2 ± 0.6^†^	1.5 × 10^3^ (0.049)*	2.9 ± 0.8	40.0 ± 5.0	1231.8 ± 111.2
Caudate	124.6 ± 25.6	5.7 ± 1.3^†^	8.6 × 10^4^ (0.029)*	2.1 ± 0.6	1.4 × 10^3^ (0.14)	2.9 ± 0.9	30.4 ± 3.8	1155.4 ± 129.0
MTL	125.3 ± 19.8	6.9 ± 1.0	1.0 × 10^4^ (0.81)	1.3 ± 0.2^†^	3.5 × 10^3^ (0.032)*	5.2 ± 0.6	38.5 ± 4.0	1187.5 ± 108.1
Amygdala	133.4 ± 24.8^†^	6.5 ± 1.6	−0.8 × 10^4^ (0.98)	1.2 ± 0.4^†^	1.6 × 10^3^ (0.25)	5.5 ± 1.4	41.0 ± 4.9	1234.0 ± 134.2
Hippocampus	124.7 ± 19.9	7.6 ± 1.2^†^	3.1 × 10^4^ (0.32)	1.1 ± 0.2^†^	3.4 × 10^3^ (0.038)*	6.6 ± 0.9	38.9 ± 4.1	1197.7 ± 116.5
PHG	123.9 ± 20.5	6.4 ± 0.9	−3.1 × 10^4^ (0.48)	1.7 ± 0.3^†^	2.4 × 10^3^ (0.08)	3.9 ± 0.5	37.7 ± 3.8	1167.6 ± 104.6

Vp and test-retest averaged CBF and ATT values were also listed. Daggers (†) indicate the brain regions with significantly different Ktrans, k_Gad_ or test/retest averaged kw than whole-brain average. P-values of mixed-effect linear regressions were listed in the parentheses. Significant correlations with P < 0.05 were marked by asterisks (*).

*MCA perf, middle cerebral artery perforator territory; MTL, medial-temporal lobe; PHG, parahippocampal gyrus; ICC, intraclass correlation coefficient*.

Average kw was 122.3 min^−1^, 122.6 min^−1^, and 121.9 min^−1^ in the whole brain, GM and WM, respectively, which were consistent with the kw values reported in recent studies (Shao et al., 2019; Anderson et al., 2020), and slightly lower than kw values (159/165 min^−1^ in GM/WM) averaged across all previously published results (Dickie et al., 2020). Average kw in amygdala (133.4 min^−1^, *P* = 0.002) was significantly higher than the whole brain average kw, as indicated by † in Table 2.

**Figure 4:**
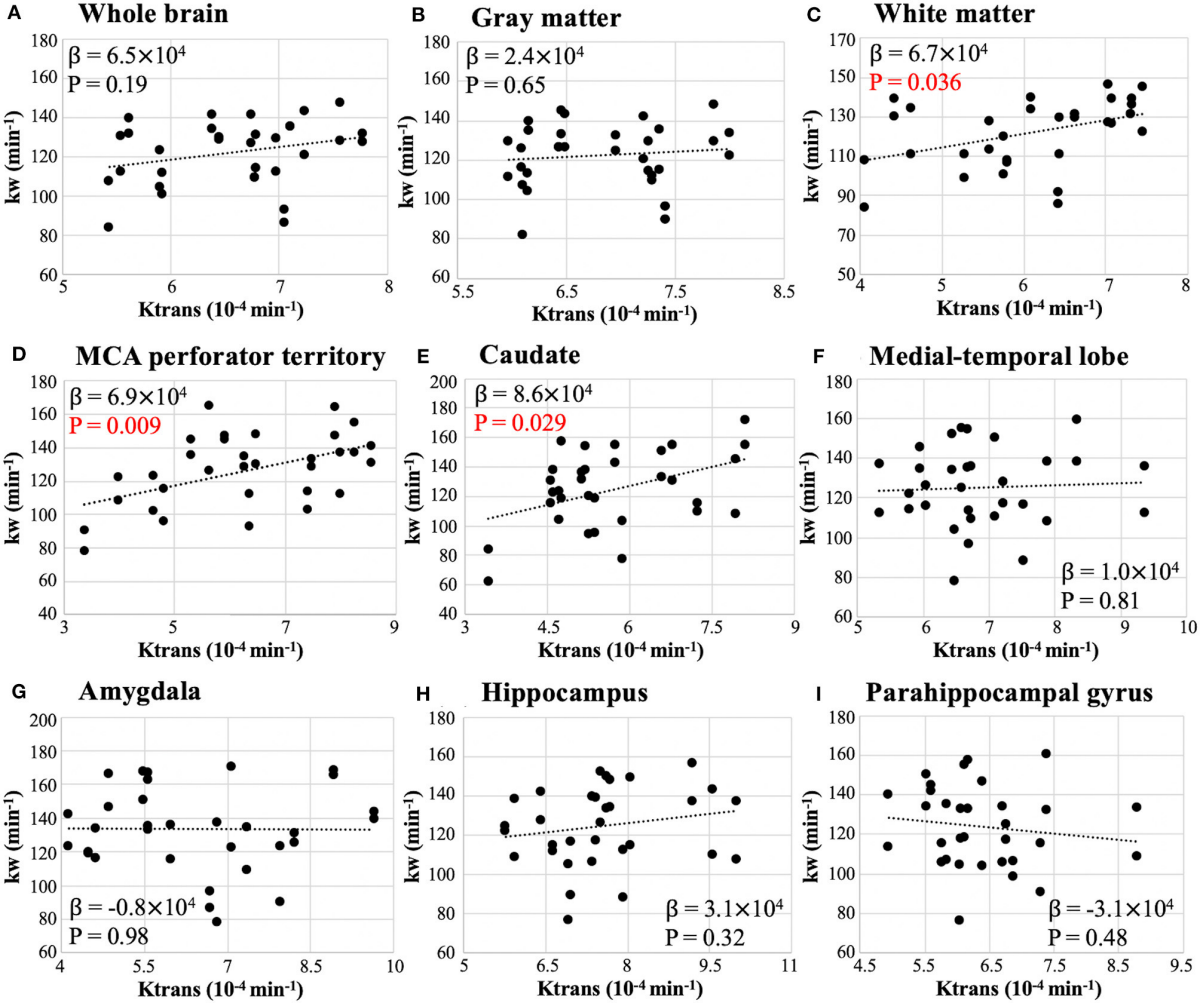
Scatter plot of whole-brain **(A)** and regional **(B–I)** Ktrans vs. kw from test and retest scans. β is the regression coefficient of the mixed-effect linear regression. Significant correlation was found in white matter (β = 6.7 × 10^4^, *P* = 0.036), caudate (β = 8.6 × 10^4^, *P* = 0.029), and MCA perforator territory (β = 6.9 × 10^4^, *P* = 0.009).

Figure 4 shows the scatter plots between whole-brain and regional test-retest kw and Ktrans values. Regression coefficients and *P*-values of mixed effect linear regressions are summarized in Table 2 (column 4). Significant correlation was found between kw and Ktrans in WM (β = 6.7 × 10^4^, *P* = 0.036), MCA perforator territory (β = 6.9 × 10^4^, *P* = 0.009), and caudate (β = 8.6 × 10^4^, *P* = 0.029), as shown in Figures 4C–E, respectively. No significant correlations were found between kw and Ktrans in the whole brain (*P* = 0.19, Figure 4A), GM (*P* = 0.65, Figure 4B), MTL (*P* = 0.81, Figure 4F), Amygdala (*P* = 0.98, Figure 4G), hippocampus (*P* = 0.32, Figure 4H), or parahippocampal gyrus (*P* = 0.48, Figure 4I).

**Figure 5:**
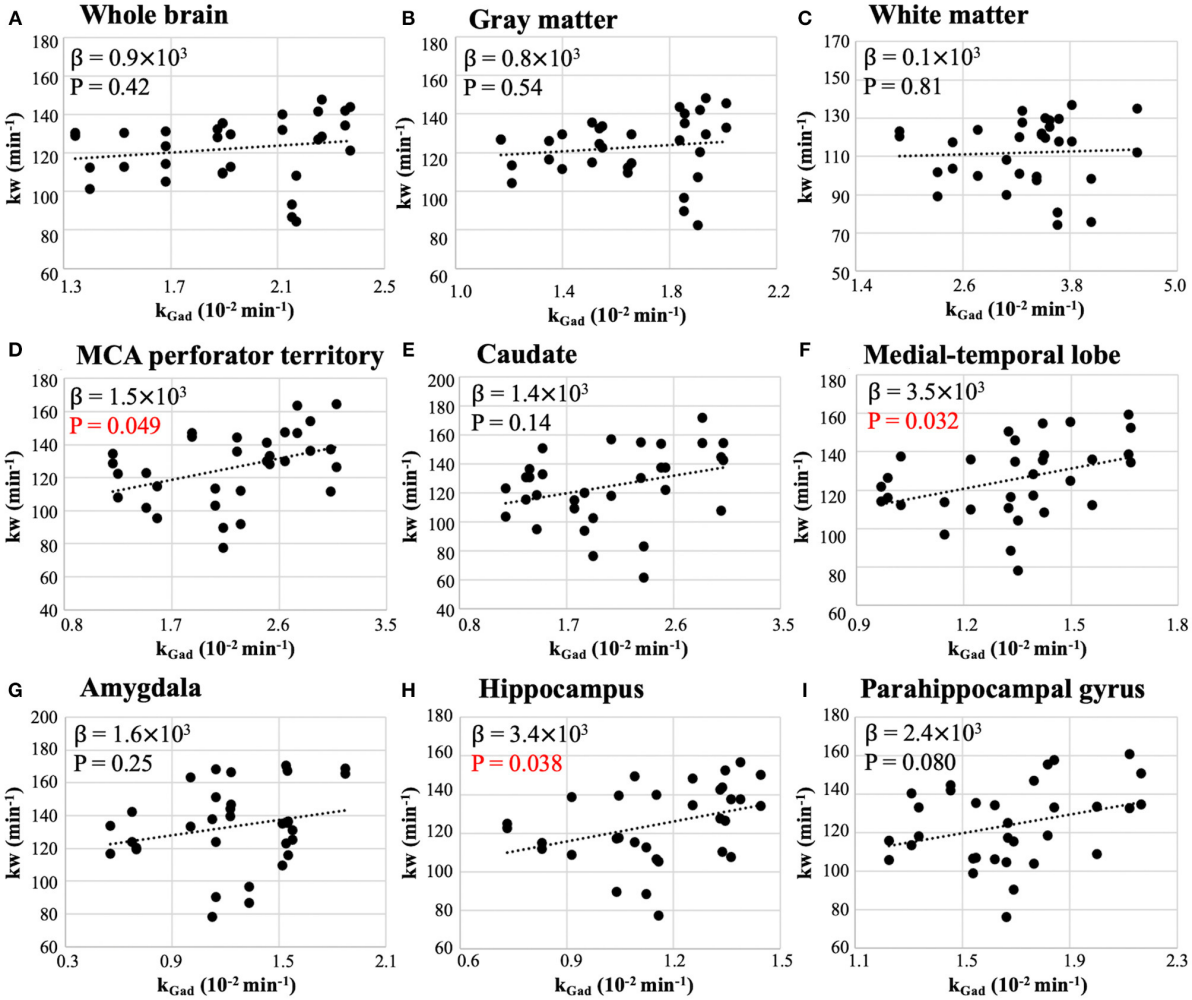
Scatter plot of whole-brain **(A)** and regional **(B–I)** k_Gad_ vs. kw from test and retest scans. β is the regression coefficient of the mixed-effect linear regression. Significant correlation was found in MCA perforator territory (β = 1.5 × 10^3^, *P* = 0.049), medial-temporal lobe (β = 3.5 × 10^3^, *P* = 0.032), and hippocampus (β = 3.4 × 10^3^, *P* = 0.038).

Figure 5 shows the scatter plots between whole-brain and regional test-retest kw and k_Gad_ values. Regression coefficients and *P*-values of mixed effect linear regressions are summarized in Table 2 (column 6). Significant correlation was found between kw and k_Gad_ in MCA perforator territory (β = 1.5 × 10^3^, *P* = 0.049), medial-temporal lobe (β = 3.5 × 10^3^, *P* = 0.032), and hippocampus (β = 3.4 × 10^3^, *P* = 0.038), as shown in Figures 5D,F,H, respectively. No significant correlations were found between kw and k_Gad_ in the whole brain (*P* = 0.42, Figure 5A), GM (*P* = 0.54, Figure 5B), WM (*P* = 0.81, Figure 5C), caudate (*P* = 0.14, Figure 5E), amygdala (*P* = 0.25, Figure 5G), or parahippocampal gyrus (*P* = 0.08, Figure 5I).

### Reproducibility of DP-pCASL Measurements

**Figure 6:**
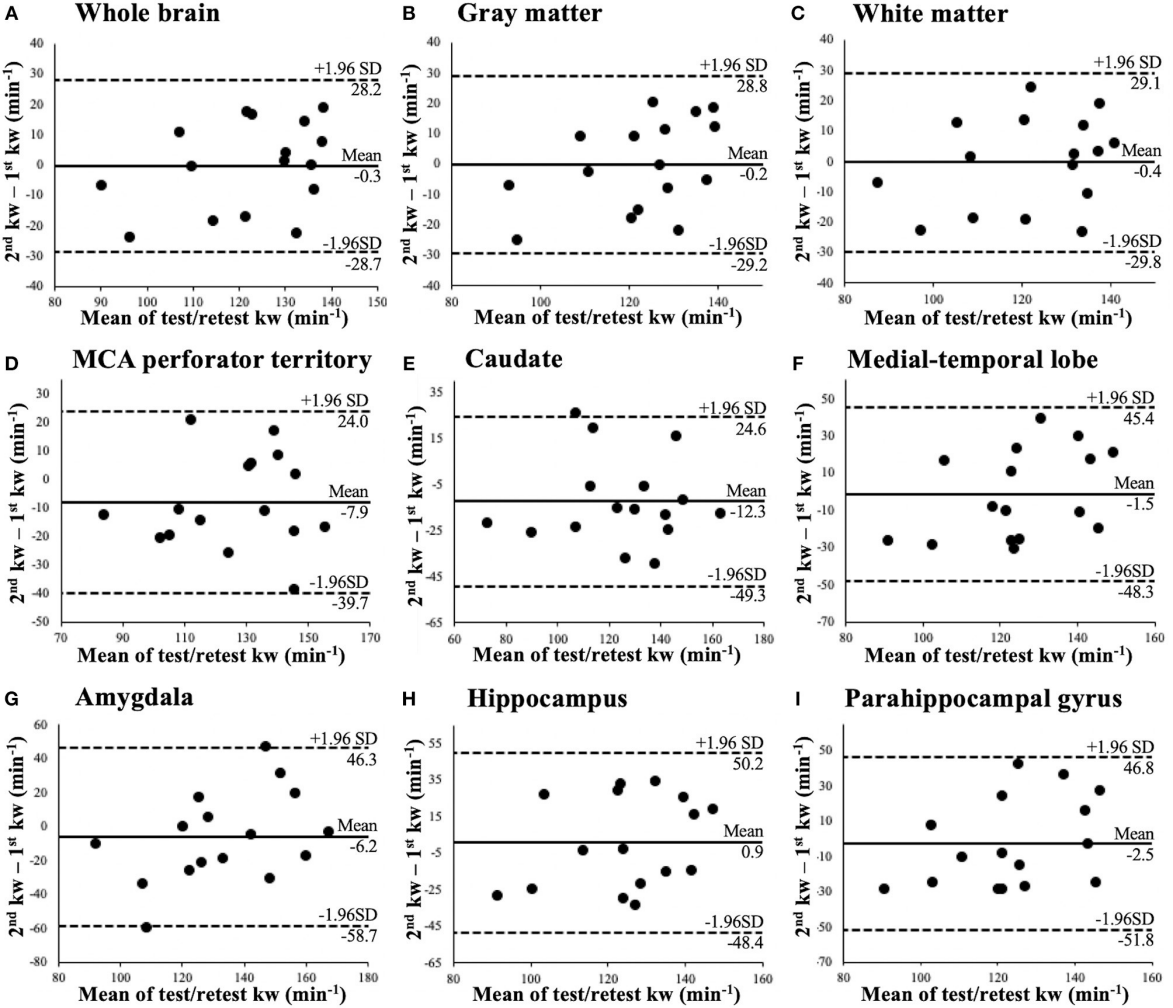
Bland-Altman plots of test and retest kw measurements in whole brain **(A)**, gray matter **(B)**, white matter **(C)**, and six subcortical regions **(D–I)**.

Table 3 summaries kw, CBF values and respective ICC measured from test and retest scans. tSNR of perfusion measurements acquired at PLD = 1,800 ms with and without diffusion preparation were also listed in Table 3. Good test-retest reproducibility was achieved for the kw measurement in whole brain (ICC = 0.75), GM (ICC = 0.74), WM (ICC = 0.79), MCA perforator territory (ICC = 0.76), and caudate (ICC = 0.79) while fair reproducibility was observed in smaller ROIs: MTL (ICC = 0.47), amygdala (ICC = 0.60), hippocampus (ICC = 0.39), and parahippocampal gyrus (ICC = 0.44). The above observation was further illustrated in Figure 6 of the Bland-Altman plots. The differences between the two repeated kw measurements had a small bias (−3.4 ± 4.5 min^−1^) with a spread (±1.96 × SD) ranging from ±28.4 min^−1^ in the whole brain (Figure 6A), ±29.0 min^−1^ in GM (Figure 6B), and ±29.5 min^−1^ in WM (Figure 6C) to ±52.5 min^−1^ in Amygdala (Figure 6G), ±49.3 min^−1^ in hippocampus (Figure 6H) and ±49.3 min^−1^ in parahippocampal gyrus (Figure 6I).

**Table 3:** Summary of kw, CBF values and intra-class correlation coefficients (ICC) from test and retest scans in the whole brain, gray matter, white matter, and six subcortical brain regions.

	**kw (min-1)**			**CBF (ml/100g/min)**			**tSNR**	
	**Visit 1**	**Visit 2**	**ICC**	**Visit 1**	**Visit 2**	**ICC**	**PLD1800 w/o DP**	**PLD1800 w/DP**
Whole brain	122.4 ± 14.3	122.2 ± 18.9	0.75	44.55.7	43.3 ± 5.1	0.88	1.11 ± 0.17	1.01 ± 0.13
Gray matter	122.7 ± 18.7	122.5 ± 13.0	0.74	45.5 ± 5.8	44.1 ± 5.2	0.89	1.13 ± 0.18	1.02 ± 0.13
White matter	122.1 ± 15.3	121.7 ± 19.3	0.79	36.8 ± 5.0	37.5 ± 4.5	0.83	0.99 ± 0.13	0.91 ± 0.10
MCA perf	130.1 ± 21.1	122.3 ± 21.9	0.76	40.4 ± 5.9	39.6 ± 5.0	0.85	1.03 ± 0.14	0.99 ± 0.12
Caudate	130.8 ± 25.5	118.5 ± 25.0	0.79	30.3 ± 4.0	30.6 ± 3.7	0.95	0.93 ± 0.13	0.91 ± 0.11
MTL	126.0 ± 15.9	124.6 ± 23.7	0.47	39.8 ± 4.8	37.2 ± 4.0	0.79	0.97 ± 0.12	0.92 ± 0.09
Amygdala	136.5 ± 19.5	130.3 ± 29.5	0.60	42.0 ± 5.8	40.0 ± 4.8	0.80	0.99 ± 0.14	0.98 ± 0.12
Hippocampus	124.3 ± 17.2	125.2 ± 22.9	0.39	39.8 ± 4.8	37.6 ± 4.1	0.78	0.99 ± 0.12	0.93 ± 0.10
PHG	125.2 ± 16.6	122.7 ± 24.3	0.44	39.3 ± 4.6	36.2 ± 3.8	0.77	0.94 ± 0.11	0.90 ± 0.09

*tSNR of perfusion measurements with and without diffusion preparation were also listed. CBF was quantified from the perfusion signals without diffusion preparation, and kw was quantified from the ratio between two perfusion measurements with TGV regularized SPA modeling*.

*MCA perf, middle cerebral artery perforator territory; MTL, medial-temporal lobe; PHG, parahippocampal gyrus; ICC, intraclass correlation coefficient*.

ICC of CBF measurements ranged from 0.77 to 0.95 from small to large brain areas, and tSNR averaged from 9 brain areas were 1.01 ± 0.07 without diffusion preparation and 0.95 ± 0.05 with diffusion preparation, respectively. Both ICC and tSNR matches well with the literature about pCASL reproducibility (ICC~0.9, tSNR~1.2) (Chen et al., 2011). ICC of kw significantly correlated with ICC of CBF measured in 9 brain areas (ρ=0.8, *P* = 0.01, Spearman correlation).

### DCE-MRI and DP-pCASL Measurements in NAWM, WMH and WMH Penumbra

**Figure 7:**
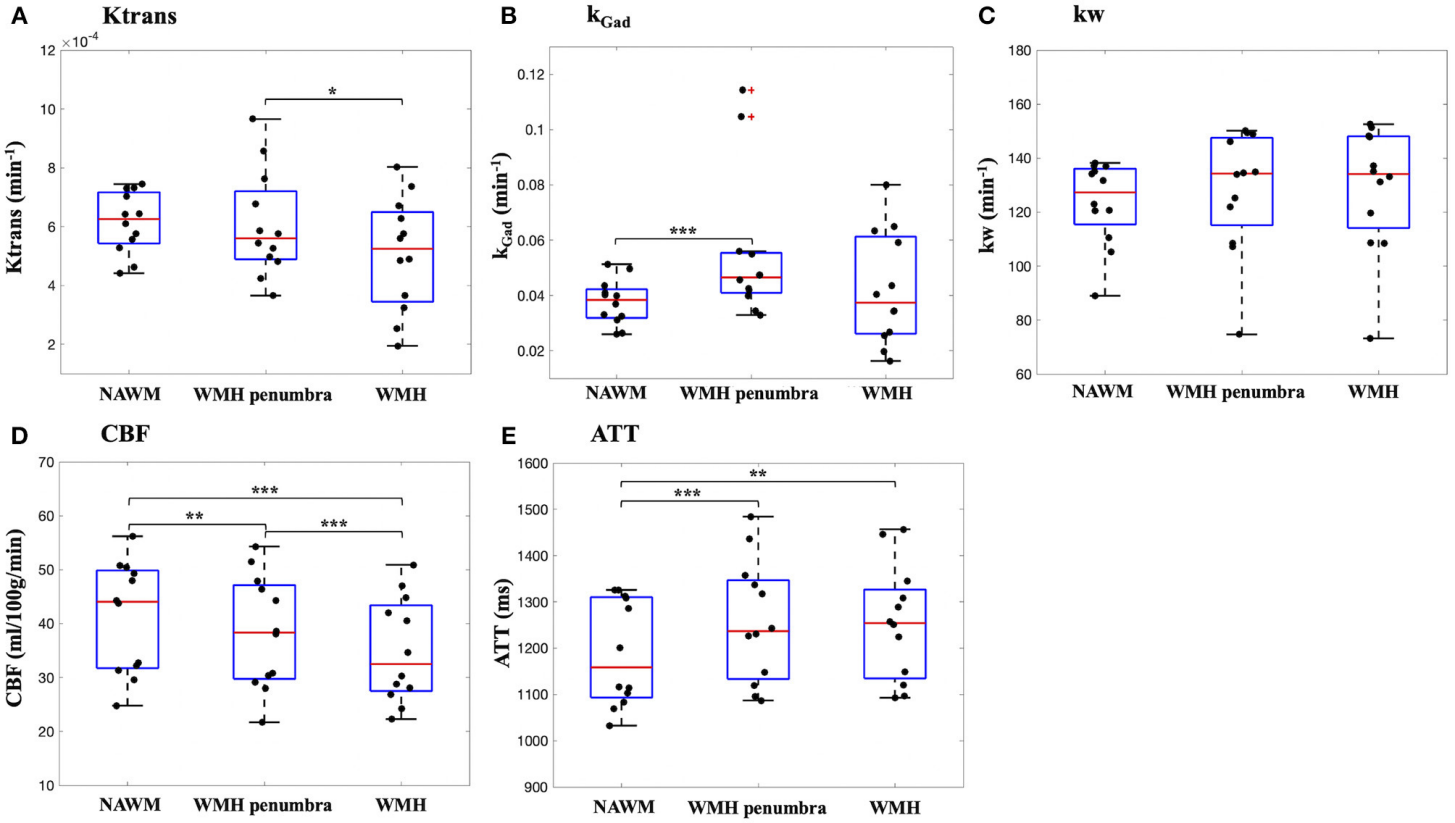
Bar plot of average Ktrans **(A)**, k_Gad_ **(B)**, kw **(C)**, CBF **(D)**, and ATT **(E)** in NAWM, WMH penumbra, and WMH. Significant difference is indicated by asterisks (*P < 0.05; **P < 0.01; ***P < 0.001).

Average WMH volume from 12 subjects were 5.4 ± 4.9 cm^3^. Table 4 summarizes the Ktrans, k_Gad_ and test-retest average kw, ATT, CBF values in NAWM, WMH, and WMH penumbra, respectively. Figure 7A shows the bar plot of Ktrans values in NAWM, WMH, and WMH penumbra. We found Ktrans was significantly lower inside WMH than in the WMH penumbra (16.2%, *P* = 0.026). No significant difference of Ktrans was found between NAWM and WMH (*P* = 0.07) or NAWH and WMH penumbra (*P* = 0.81). Figure 7B shows the bar plot of k_Gad_ values in NAWM, WMH, and WMH penumbra. Two subjects with extremely high k_Gad_ in WMH penumbra were considered as outliers (red cross in Figure 7B) and excluded for comparison. k_Gad_ was significantly lower in NAWM than in the WMH penumbra (20.8%, *P* < 0.001). No significant difference of Ktrans was found between NAWM and WMH (*P* = 0.46) or WMH and WMH penumbra (*P* = 0.45). Figure 7C shows the bar plot of 2-visit averaged kw values in NAWM, WMH and WMH penumbra. No significant difference of kw was found between NAWM and WMH (*P* = 0.09), NAWM and WMH penumbra (*P* = 0.10), or WMH and MMH penumbra (*P* = 0.22). Eight out of these 12 subjects had more than one WMHs and WMH penumbras. Average and standard deviation of Ktrans, kGad, and kw in multiple WMHs and WMH penumbras are summarized in Table 5. We found Ktrans and k_Gad_ had larger spread across WMH and WMH penumbras in each subject. Standard deviation of Ktrans, k_Gad_ and kw across multiple WMHs were 74.3 ± 44.2%, 88.1 ± 20.1%, and 20.2 ± 13.8% of respective values averaged in all WMHs; Standard deviation of Ktrans, k_Gad_, and kw between multiple WMH penumbras were 56.1 ± 21.7%, 54.0 ± 30.9%, and 17.9 ± 10.2% of respective values averaged in all WMH penumbras across 8 subjects, respectively. We found average Ktrans and kGad in WMHs were significantly correlated (linear regression) with the number of WMHs (Ktrans: β = 1.1 × 10^−5^, *P* = 0.02; kGad: β = 1.3 × 10^−3^, *P* = 0.02), and observed a positive trend between WMH kw and the number of WMHs (β = 1.2, *P* = 0.18).

**Table 4:** Summary of Ktrans and test-retest kw, CBF and ATT values in NAWM, WMH and WMH penumbra from 12 subjects with WMH size >1 cm^3^.

	**Ktrans (×10–4 min^**−1**^)**	**k_**Gad**_ (×10^**−2**^ min^**−1**^)**	**kw (min-1)**	**CBF (ml/100g/min)**	**ATT (ms)**
NAWM	6.1 ± 1.0	3.5 ± 0.6	123.5 ± 16.9	41.1 ± 5.2	1189.8 ± 114.7
WMH penumbra	6.1 ± 1.8	4.3 ± 0.8	128.0 ± 23.5	38.4 ± 5.2	1256.8 ± 132.0
WMH	5.1 ± 1.9	4.0 ± 2.1	128.9 ± 24.5	35.0 ± 4.8	1253.1 ± 124.7

*NAWM, normal appearing white matter; WMH, white matter hyperintensity*.

**Table 5:** Summary of average, SD and spread (SD/average) of Ktrans, k_Gad_ and kw values in multiple WMHs (top) and WMH penumbras (bottom).

**Ktrans, k**_****Gad****_ **and kw in multiple WMHs**										
**Subject #**	**Number of WMHs**	**Ktrans (10**^**−4**^ **min**^**−1**^**)**			**k**_**Gad**_ **(10**^**−2**^ **min**^**−1**^**)**			**kw (min**^**−1**^**)**		
		**Average**	**SD**	**SD/average**	**Average**	**SD**	**SD/average**	**Average**	**SD**	**SD/average**
1	36	8.0	4.2	0.52	6.3	6.2	0.98	148.3	15.8	0.11
2	6	5.8	6.0	1.04	3.4	2.5	0.74	147.9	20.6	0.14
3	3	3.7	5.9	1.61	4.4	2.5	0.57	135.1	18.8	0.14
4	8	2.5	0.4	0.17	1.6	1.3	0.81	108.6	19.8	0.18
5	23	6.3	2.3	0.36	8.0	8.4	1.05	151.3	34.9	0.23
6	23	5.6	4.3	0.77	5.9	7.2	1.22	133.1	19.8	0.15
7	4	4.9	3.4	0.69	3.4	3.0	0.88	73.2	38.8	0.53
8	11	4.8	3.8	0.78	2.0	1.6	0.80	119.6	15.4	0.13
**Ktrans, k**_**Gad**_ **and kw in multiple WMH penumbras**										
**Subject #**	**Number of WMH penumbras**	**Ktrans (10**^**−4**^ **min**^**−1**^**)**			**k**_**Gad**_ **(10**^**−2**^ **min**^**−1**^**)**			**kw (min**^**−1**^**)**		
		**Average**	**SD**	**SD/average**	**Average**	**SD**	**SD/average**	**Average**	**SD**	**SD/average**
1	19	8.6	3.5	0.41	4.7	1.9	0.40	146.2	13.5	0.09
2	3	6.8	3.2	0.47	4.6	0.9	0.20	148.9	16.9	0.11
3	3	5.9	4.6	0.78	11.4	7.1	0.62	134.5	22.6	0.17
4	8	5.0	2.6	0.53	3.3	1.5	0.45	107.2	18.1	0.17
5	15	5.8	2.8	0.49	4.8	2.7	0.56	150.2	30.8	0.21
6	13	4.8	3.1	0.64	5.6	6.4	1.14	134.9	11.6	0.09
7	4	5.3	1.3	0.24	5.5	1.1	0.20	74.7	30.3	0.41
8	11	3.7	3.4	0.93	3.4	2.5	0.74	121.9	23.3	0.19

*Eight subjects had more than one WMHs and WMH penumbras. Number of WMHs and WMH penumbras identified from each subject's FLAIR image were listed in the second column. Number of WMH penumbras may be smaller than number of WMHs because two or more WMH lesions can be surrounded by the same penumbra. Average value was calculated from all WMHs or penumbras, and SD was calculated as the standard deviation of Ktrans/k_Gad_/kw values averaged in each WMH/penumbra (SD/average can be <1). Ktrans and k_Gad_ had larger spread across WMH and WMH penumbras. Average spread of Ktrans, k_Gad_, and kw across multiple WMHs were 74.3 ± 44.2%, 88.1 ± 20.1%, and 20.2 ± 13.8%; Average spread of Ktrans, k_Gad_, and kw between multiple WMH penumbras were 56.1 ± 21.7%, 54.0 ± 30.9%, and 17.9 ± 10.2%, respectively*.

*SD, standard deviation*.

**Figure 8:**
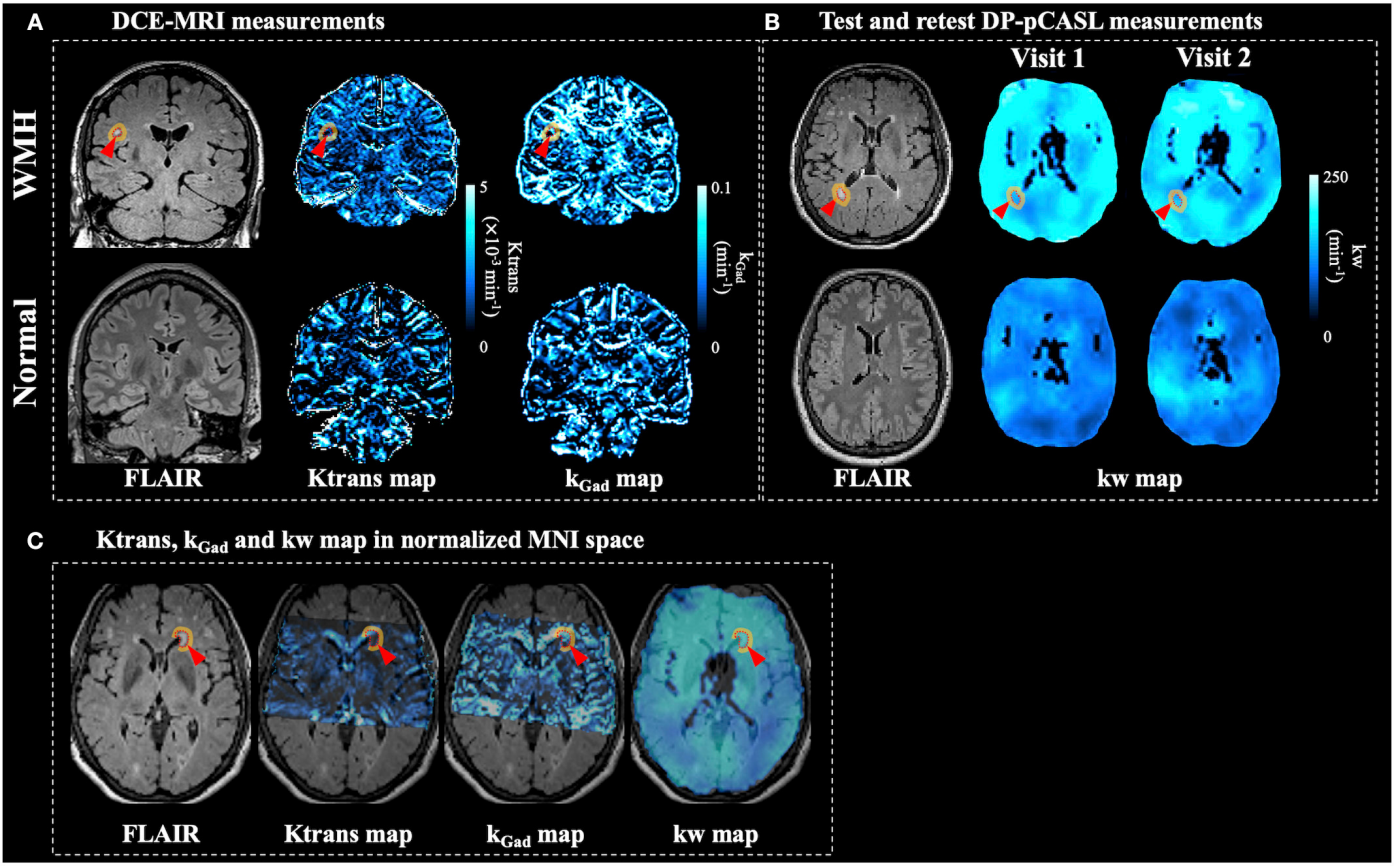
Ktrans and k_Gad_ **(A)** and test-retest kw **(B)** maps from subjects with (top row) and without (bottom row) WMH. **(C)** FLAIR, Ktrans, k_Gad_, and kw maps (first visit) in normalized MNI space from the same subject as shown in the top row. Ktrans, k_Gad_, and kw maps were overlaid on the FLAIR image. One WMH lesion was indicated by a red arrow and a dashed red circle and WMH penumbra was indicated by an orange circle on each image.

Figure 8A shows one coronal slice of FLAIR, Ktrans and k_Gad_ images from two representative subjects with and without WMH, respectively, and Figure 8B shows one axial slice of the FLAIR and kw images from test and retest scans from the same subjects. Figure 8C shows Ktrans, k_Gad_, and kw map in normalized MNI space for comparison between DCE-MRI and DP-pCASL measurements in the same orientation. WMH was indicated by a red arrow and dashed red circle, and WMH penumbra was indicated by an orange circle on each image. Good agreement of kw maps was observed between test and retest scans, which indicates overall high reliability of the kw measurement using the proposed DP-pCASL sequence despite some variations in smaller ROIs. In-plane spatial resolution of Ktrans and k_Gad_ maps was higher, which allowed the detection of permeability variations in white matter lesions and surrounding penumbra. The spatial coverage of DCE-MRI was not full brain (70 mm in anterior-posterior direction) because this protocol was designed to cover MTL and study BBB permeability in brain areas associated with cognitive impairment and cSVD.

Figures 7D,E show the bar plot of 2-visit averaged CBF and ATT values in NAWM, WMH and WMH penumbra, respectively. CBF in NAWM was significantly higher than CBF in WMH penumbra (7.0%, *P* = 0.006) and WMH (17.4%, *P* < 0.001). Also CBF in WMH penumbra was significantly higher than WMH (9.7%, *P* < 0.001). ATT in NAWM was significantly lower than ATT in WMH penumbra (5.4%, *P* < 0.001) and WMH (5.1%, *P* = 0.002). No significant difference of ATT was found between WMH penumbra and WMH (*P* = 0.52).

## Discussion

In this study, we measured water exchange rate across the BBB with DP-pCASL in a cohort of older subjects at risk of cSVD. To the best of our knowledge, this is the first time that water exchange rate across the BBB was directly compared with BBB permeability to GBCAs. Regional analysis was performed to investigate potential correlations between the two BBB measurements. Ktrans/k_Gad_ and kw values were also compared between NAWM, WMH, and WMH penumbra.

DCE-MRI is the most commonly used approach to measure the paracellular leakage of the GBCAs and to locate disrupted BBB when damage to the tight junctions or endothelial membrane is severe, such as in brain tumor (Heye et al., 2014). DCE-MRI has also been used to detect more subtle BBB breakdown related to aging or neurodegenerative diseases (Wardlaw et al., 2003; Farrall and Wardlaw, 2009; Montagne et al., 2015; Barnes et al., 2016; Zhang et al., 2017, 2019; Nation et al., 2019; Anderson et al., 2020). The Patlak model was found to be the most reliable kinetic model for low permeability data (Montagne et al., 2015; Barnes et al., 2016; Heye et al., 2016). However, the small change of dynamic signals still makes the estimated Ktrans sensitive to signal drift and subject motion during the long scan time (Heye et al., 2014, 2016). We found overall Ktrans is <1 × 10^−3^ min^−1^, which indicates subtle BBB leakage in the recruited subjects. The highest BBB leakage (Ktrans) was found in the hippocampus, which is 15.2% higher than the whole brain average value (*P* = 0.0002). This result is consistent with the previous finding that BBB breakdown begins in the hippocampus of the aging human brain, which may contribute to cognitive impairment (Montagne et al., 2015). Under limited permeability condition as in this study, Ktrans approximately equals to PS (Cuenod and Balvay, 2013). For a more direct comparison with kw, we calculated the exchange rate of GBCA (k_Gad_) as the ratio between Ktrans and Vp. Both k_Gad_ and kw are insensitive to the fractional blood volume.

The growing research interests in trans-BBB water exchange measurements have been mainly driven by the need for studying subtle BBB alterations. Given that water molecules are much smaller than the GBCAs and transvascular water exchange is mediated by passive diffusion and active cotransport through endothelial membrane and water-selective facilitated diffusion through AQP4 at end feet of astrocytes (Ibata et al., 2011; Papadopoulos and Verkman, 2013), assessing kw could potentially provide a more direct and sensitive assessment of BBB dysfunction at an earlier stage of a wide range of BBB pathologies. ASL has been employed to track the transcapillary exchange of labeled water as a contrast-free technique. Precise and accurate separation of the 2 components of the ASL signal is critically important for reliable water exchange measurement. In this study we first measured ATT using the FEAST method (1183.0 and 1230.7 ms in GM and WM) (Wang et al., 2003), and incorporated ATT in kw quantification. Perfusion signal acquired at PLD of 1,800, when majority of labeled water arrives at the capillary space and exchanges with tissue, was used for kw measurement. Although PLD longer than 1,800 ms (i.e., 2,000–2,500 ms) are sometimes used for more accurate WM perfusion mapping, longer PLD may make it challenging for or even violate the SPA modeling for kw quantification, as most of the label will reside within the extravascular compartment, and efflux of label from the extravascular compartment may equal or be greater than the influx (Dickie et al., 2020). Similarly, our choice of labeling duration (1,500 ms) was based on an optimal tradeoff between SNR and SAR efficiency. Considering the pseudo-diffusion coefficient of capillary water is ~100-fold higher than that of the tissue (Shao et al., 2019), the proposed DP-pCASL applies a small diffusion gradient (50 s/mm^2^) so that the vascular component of the ASL can be reliably distinguished from tissue signal. Reliability of the kw measurement was further improved by TGV regularized SPA modeling. Reproducibility of kw was evaluated by test and retest scans (~6 weeks apart) of the same subject. Good reproducibility (ICC, 0.74–0.79) was found in large ROIs (whole brain, GM, WM, MCA perf) as well as in caudate (Koo and Li, 2016). Poor to moderate reproducibility (ICC, ~0.4–0.6) of kw was observed in smaller regions of the MTL (amygdala, hippocampus and parahippocampal gyrus). *In vivo* water exchange studies started with measuring the water permeability-surface area product (PSw, ml/100g/min) using ${\text{H}}_{2}^{15}$O PET (Herscovitch et al., 1987). Compared to PSw, the water exchange rate, kw (min^−1^), measured by DP-pCASL, is less affected by the surface area of the microvessels. For instance, we found no significant difference between GM and WM kw (*P* = 0.62, paired *t*-test) in this study, which is consistent with a recent review that summarized all previously published results (Dickie et al., 2020), while a previous study reported ~85% higher PSw in GM than WM (Rooney et al., 2015). This PSw difference may be largely attributed to the 67.3% larger capillary surface areas in GM (Schlageter et al., 1999).

Both kw and Ktrans/k_Gad_ directly measure the function and integrity of BBB although they assess different transport mechanisms of water and GBCA, respectively. The tight junctions of the endothelial cells restrict the free paracellular diffusion of GBCAs from capillary to brain tissue, and GBCAs leakage is slow until BBB opening reaches a certain critical level (Nitta et al., 2003). Water exchange across the BBB is mediated by multiple pathways including the dedicated water channel AQP4, and redistribution of AQP4 has been associated with pericyte deficiency at the early stage of BBB disruption (Warth et al., 2004; Montagne et al., 2018), as well as with the accumulation of amyloid deposits in AD (Yang et al., 2011). Additionally, water is an abundant endogenous tracer, and water exchange rate (122.3 min^−1^) is ~6,000 × faster than the exchange rate of the GBCAs across the BBB (1.9 × 10^−2^ min^−1^), suggesting that kw changes are more likely to be detected at an earlier stage when BBB leakage is subtle.

BBB integrity relies on tight junction proteins (Nitta et al., 2003) as well as the polarity of astrocytes, which is maintained by AQP4 on astrocyte end-foot membranes (Yang et al., 2011; Ohene et al., 2019). Thus, one hypothesis is that associations between Ktrans, k_Gad_, and kw are likely to be detected under certain pathological conditions (i.e., loss of tight junction) which drive the Ktrans/k_Gad_ and kw to change toward the same direction.

We found significant positive correlations between kw and Ktrans in WM, MCA perforator territory. These results are consistent with literature evidence that the microvasculature and BBB in WM and MCA perforator territory are more susceptible to vascular impairment caused by cSVD compared to other cortical areas: The vasculature supplying deep WM has a long path and is the junction between several vascular territories, thus cerebral small vessel endothelial cells in WM is likely to be damaged in cSVD which may result in occurrence of WMH lesions (Taheri et al., 2011). Previous studies also reported that BBB permeability in WM increased in patients with cSVD and vascular cognitive impairment (Taheri et al., 2011; Zhang et al., 2017); The MCA perforator territory are fed by the lenticulostriate arteries, which are end arteries with almost no collaterals that could compensate for impaired perfusion due to cSVD. The tortuosity of lenticulostriate arteries may increase and subsequently contribute to the increased blood pressure and altered BBB permeability in aged subjects at risk of cSVD (Ma et al., 2019). Studies also reported that BBB integrity of small perforating arteries has been commonly affected in arteriolosclerosis, a prevalent form of cSVD (Rosenberg et al., 2016). Besides WM and MCA perforator territory, we also observed a significant correlation between kw and Ktrans in caudate, a critical deep GM nucleus involved with storing and processing memories. A previous study also reported that BBB dysfunction in caudate is associated with early pathological changes in cSVD with minimal AD pathology (Bridges et al., 2014). Increased BBB leakage to GBCA and water exchange rate in the WM, MCA perf territory and caudate may indicate vascular impairment caused by cSVD in this aged cohort. This result suggests that both kw and Ktrans are sensitive to BBB dysfunction, which are correlated only in brain regions that are most susceptible to vascular impairment caused by cSVD. However, the underlying mechanisms of kw and Ktrans are likely to be different, therefore no significant correlations were observed in the rest brain regions studied.

We also observed significant positive correlations between kw and k_Gad_ in MCA perforator territory, hippocampus and medial-temporal lobe. Medial temporal lobe including the hippocampus is a critical structure for Alzheimer's disease related pathology, and it has been reported that early BBB breakdown influences cognitive function mostly in the medial temporal lobe (Montagne et al., 2015; Nation et al., 2019). Both kw and k_Gad_ measures the exchange rate of water and GBCA across the BBB, respectively, controlling for potential changes of vascular volume under pathological conditions. It is worth noting that k_Gad_ was not commonly used for GBCA permeability studies, therefore this finding need to be interpreted with caution and replicated in future studies with larger sample size.

Extensive WMH lesions are usually considered as a surrogate marker of cSVD disease severity (Farrall and Wardlaw, 2009; Zhang et al., 2019). However, researches on WMH related Ktrans BBB permeability changes are not consistent. Starr et al. reported increased Ktrans in subjects with higher white matter lesion scores (Starr et al., 2003), while Zhang et al. reported larger WMH volume is associated with lower Ktrans in lesion regions and no association was found in normal appearing white matter nor gray matter (Zhang et al., 2019). It is possible that BBB disruption precedes the development of visible WMH and therefore the association between Ktrans and WMH depends highly on the stage of disease progression and the degree of BBB disruption. For example, Huisa et al. reported markedly reduced permeability inside the WMH, while the majority of the increased permeability was found in the surround penumbra (Huisa et al., 2015). In our study, we also found Ktrans in WMH penumbra is significantly higher than Ktrans within the WMH and k_Gad_ in WMH penumbra is significantly higher than k_Gad_ in NAWM, which indicates that Ktrans and k_Gad_ images have sufficient in-plane spatial resolution to detect BBB permeability variations in small ROIs. Ktrans and k_Gad_ also have larger spread between WMHs (74.3 and 88.1%) and WMH penumbras (56.1 and 54.0%) in each subject as compared to kw (~20%). We found average Ktrans and k_Gad_ in WMHs were significantly correlated with the number of WMH lesions, which indicates BBB leakage (to GBCAs) within WMH increases along with WMH lesion development. The reason for the larger spread of DCE-MRI measurements across WMH and WMH penumbras compared to that of DP-pCASL is not clear, and may reflect different stages of WMH progression and/or different imaging resolutions between DCE and DP-pCASL MRI. No significant difference of kw was found between three WMH related regions, which is consistent with a recent study that no significant kw difference was found between progressive and non-progressive WMH ROIs although variations of kw in progressive ROIs were larger (Fujima et al., 2020). Additionally, we observed significantly different CBF and ATT (same resolution as kw) in 3 WMH related regions, which suggests that current imaging protocol has sufficient sensitivity to detect physiological variations between small ROIs. *One* major limitation of the current kw measurement is the relatively lower spatial resolution, which may affect the accuracy of kw measurements and sensitivity for detecting kw changes in smaller ROIs (i.e., WMH). Additionally, partial volume effect and blurring along slice direction due to long echo train length could aggravate the situation. Currently DCE-MRI provides higher spatial information and can be used to study BBB leakage in small brain areas such as hippocampus (Montagne et al., 2015), and kw measurement with the existing protocol cannot replace DCE-MRI to study BBB leakage in small brain regions which require high spatial resolution. For kw quantification in this study, voxels with large signal fluctuation across measurements were removed, which were likely to reside in large arteries or ventricles. WM masks were also eroded to avoid partial volume effect between CSF or GM. To improve the spatial resolution and sensitivity to detect kw changes in small ROIs, accelerated single-shot GRASE sequence and spatio-temporal regularized reconstruction might be utilized (Spann et al., 2020).

There are other limitations of this study. First, the sample size of the study is relatively small and consisted of 100% elderly Latinx subjects. This and other factors such as MRI scanner difference may explain the lower WM Ktrans measured in this study compared to earlier studies in larger cohorts (Montagne et al., 2015, 2016; Nation et al., 2019). Future studies with a larger sample size of diverse populations and longitudinal scans are desirable to investigate the Ktrans and kw change at different stages of disease progression. Second, limited by the small sample size, we did not study the association between the two BBB measurements and vascular risk factors or cognitive performance, which should be evaluated in future studies on larger cohorts. Third, several groups proposed measuring water exchange using DCE-MRI. Rooney et al. proposed measuring water exchange rate with dynamic analysis of the first-pass signal of shutter-speed DCE-MRI (Rooney et al., 2015). Direct comparison between the water exchange rate measured by DCE-MRI and DP-pCASL would be desirable. However, higher temporal resolution (~ 2 secs/frame) (Rooney et al., 2015) is required to fully capture the first-pass dynamics using method provided by Rooney et al. Our DCE-MRI protocol was designed for Ktrans measurement with longer acquisition time, therefore insufficient time points were acquired within the first-pass window despite our fairly high temporal resolution (15 secs/frame) compared to other DCE-MRI studies (Wardlaw et al., 2003; Farrall and Wardlaw, 2009). And a slower injection rate can be useful to capture the first pass peak to assess water exchange (Rooney et al., 2015). Dickie et al. proposed measuring water exchange using multiple flip angle after the first-pass peak (Dickie et al., 2019). This technique provides whole brain coverage and does not require high temporal resolution. However, this technique may require a relatively long pre-scan for flip-angle error calibration and pre-contrast T1 mapping.

In conclusion, water exchange rate across the BBB was compared with Ktrans and k_Gad_ in a cohort of aged subjects at risk of cSVD. A positive correlation was found between kw and Ktrans in the WM, MCA perforator territory and caudate, and between kw and k_Gad_ in MCA perforator territory, hippocampus and medial-temporal lobe, but not in the rest regions. kw provides a measure of water exchange rate across the BBB with good test-retest reproducibility. Compared to DP-pCASL, DCE-MRI provides higher spatial information. Higher Ktrans was found in WMH penumbra than inside WMH and higher k_Gad_ was found in WMH penumbra than NAWM, while no significant difference of kw was found between WMH penumbra and WMH regions.

## Data Availability Statement

To request sample data as well as a standalone toolbox for kw calculation, please contact the corresponding author or submit request on our lab website (loft-lab.org) under tab “software”. Data and the reconstruction toolbox will be available after we establish Material Transfer Agreement (MTA) between user's institute and University of Southern California.

## Ethics Statement

The studies involving human participants were reviewed and approved by RoseAnn Fleming, Interim Director, University of Southern California (USC) Institutional Review Board. The patients/participants provided their written informed consent to participate in this study.

## Author Contributions

XS developed the post-processing pipeline, conducted data analysis, and wrote the manuscript. XS, KJ, SM, LY, and DW carried out the experiments and collected data. KJ, SM, and AM conducted data analysis. KJ, LY, AM, JR, BZ, and DW guided experiments, discussed results, and revised manuscript. All authors edited and revised the manuscript and approved final submission.

## Conflict of Interest

The authors declare that the research was conducted in the absence of any commercial or financial relationships that could be construed as a potential conflict of interest.
